# Combined Channel Estimation with Interference Suppression in CPSS

**DOI:** 10.3390/s18113823

**Published:** 2018-11-08

**Authors:** Xiaoyang Lai, Huan Wang

**Affiliations:** 1 School of Computer and Communication Engineering, University of Science and Technology Beijing, Beijing 100083, China; laixiaoyang@dtlinktech.com; 2 Datang Linktech Infosystem Co., Ltd, Beijing 100191, China; 3 Industry Department, China Electronics Technology Group Corporation, Beijing 100846, China

**Keywords:** channel estimation, interference suppression, noise cancellation, machine learning, K-means, KNN

## Abstract

With social characteristics integrated into cyber-physical systems (CPS), the wireless channel has been a complex electromagnetic environment due to the subjectivity of human behaviour. For the low-power and resource-constrained nodes in cyber-physical-social systems (CPSS), minimum research is available focusing on conquering the issues of computational complexity, external interference and transmission fading simultaneously. This study aims to explore channel estimation with interference suppression based on machine learning. A novel channel estimation scheme is proposed, which combined interference suppression in channel impulse response (CIR) of frequency domain with *K*-means algorithm and noise cancellation in CIR of time domain with *K*-nearest neighbor (KNN) algorithm into an integrated process. Complexity analysis and simulation results showed that the proposed scheme has relatively lower complexity and the performance is proven better than traditional schemes, which meets the requirements of CPSS in complex electromagnetic environments.

## 1. Introduction

The cyber-physical system (CPS) was coined to describe the mapping, interaction and collaboration between computational and physical resources [1]. With the tight junction between human behaviour and CPS, social characteristics integrated into CPS, which is called a cyber-physical-social system (CPSS) [2]. CPSS indicates the integration of cyber interactions, physical perceptions and social connections. Due to the subjectivity of human behaviour, the wireless channel has been a complex electromagnetic environment of mixed radio signals between human-to-human and human-to-object [3]. Under the wireless connectivity of all things, the signal types are diverse, and not only the ambient noise is increased, but also the mutual interference will appear. Especially in the connection in multimedia interaction, the signal has a broad bandwidth which is easily interrupted. It leads to short communication distance, low transmission rate and even information coverage hole in electromagnetic interference [4]. There is an interesting example: when you enter a WiFi coverage room with a Bluetooth headset, the transmission rate of WiFi will decrease because of the interference by Bluetooth [5].

Channel estimation is used for the recognition of fading characteristics in communication channel. For broadband communication, channel estimation is often realized by inserting training sequences of signals in time domain and frequency domain. The transform domain signal processing theory has achieved a good compromise between performance and complexity for channel estimation, which can then be applied in broadband communication [6]. Studies by Edford proposed to cancel the noise in the channel impulse response (CIR). of time domain, which was transformed by least square (LS) channel estimation in frequency domain [7]. Studies by Minn took two times of samples than that in the largest multipath delay, while the other samples in CIR were set to zero and treated as noise [8]. Studies by Fukuhara proposed taking the samples in the largest multipath delay in CIR. The other samples in CIR are set to zero which are treated as noise [9]. Studies by Fan presented the leakage of CIR in the practical Orthogonal Frequency Division Multiplexing (OFDM) systems, the noise existed during signal multipath delay, the frequency domain window was used to reduce the leakage of CIR, and the noise of CIR was detected by threshold [10]. With the development of machine learning in recent years, Fan and Ma have put forward discriminant analysis, clustering analysis in CIR. Through the machine learning of the distance between the samples in CIR, the clustering of noise and the distinction of signal taps in time domain were realized [11,12].

There are two typical strategies for anti-interference. One is to pre-process before transmitting, such as transmission power allocation, spectrum sharing or reusing [13,14,15]. Pre-processing is used to avoid the intended interference signal. The other strategy for anti-interference is to suppress or cancel interference by recognition of the receiving signal feature. Interference suppression or cancellation aims at uncertain interference signal. The technologies of time domain prediction, code-aided and transform domain for interference suppression are summarized in [16]. The algorithm based on time domain prediction takes advantage of the pseudo-randomness of spread spectrum signal, which has a slow convergence rate. In addition, the code-aided algorithm is based on a pseudo-random sequence with high computational complexity. The transform domain interference suppression is simple in implementation and available in application, which can fulfill the demand of CPSS for energy efficient [17].

Due to the interference in suppression and channel estimation aimed at different objects, the technologies evolved through relatively independent roadmap. While the channel estimation and interference suppression are implemented separately, the computational complexity is linear stacking in the signal detection, which increases the processing delay and the cost of hardware. Although there is some research named joint interference suppression and channel estimation, the technical relevance of conquering the two issues is not remarkable [18,19,20]. For the low-power and resource-constrained nodes in CPSS, few of the existing approaches can sufficiently conquer the issues of computational complexity, narrowband interference (NBI) and transmission fading simultaneously [21]. Inspired by the machine learning technology for channel estimation, the study aims to integrate interference suppression into channel estimation based on machine learning.

The rest of this manuscript is organized as follows: Section 2 presents the signal model in complex electromagnetic environment. Section 3 proposes the scheme of channel estimation with interference suppression. Section 4 describes the complexity analysis as well as the simulation results. Section 5 presents the application of the proposed scheme. The conclusions and future work are presented in Section 6.

## 2. Signal Model

**Figure 1:**
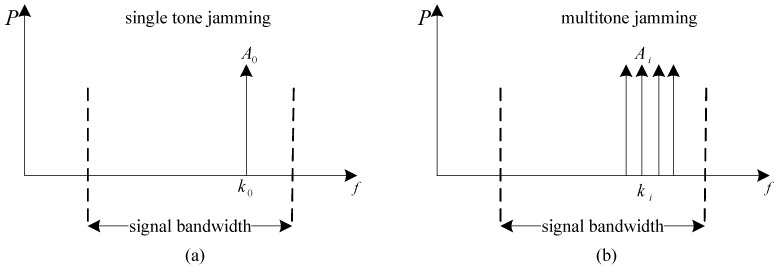
Power spectrum of NBI. (**a**) single tone jamming; (**b**) multitone jamming.

NBI is partial band jamming, which consists of a simple unmodulated carrier [22]. Figure 1a is a frequency domain monopulse, which refers to single tone jamming. The mathematical model of single tone jamming is:

(1) $$J\left(k_{I},l\right)=A_{0}\delta \left(k_{I}-k_{0},l\right).$$

Figure 1b refers to multitone jamming, which is a set of single tones. The mathematical model of multitone jamming is:

(2) $$J\left(k_{I},l\right)=\frac{1}{\sqrt{K}}\sum_{i=1}^{K}A_{i}\delta \left(k_{i}-k_{0},l\right),$$

where *K* is the number of interfered subcarrier.

Thereby, the signal with interference at the receiver can be written as:

(3) $$Y\left(k,l\right)=H\left(k,l\right)X\left(k,l\right)+J\left(k_{I},l\right)+W\left(k,l\right),$$

where H(k,l) is the CIR in frequency domain. $J(k_{I},l)$ refers to the interference with $k_{I}$ subcarriers jamming. W(k,l) is additive white Gaussian noise (AWGN). X(k,l) is a training sequence based on Zadoff–Chu (ZC) sequence, the modulus of which is 1. The Flourier transformation of ZC sequence is still a ZC sequence.

To estimate CIR H(k,l) more accurately, $J(k_{I},l)$ and W(k,l) in Equation (3) should be exactly eliminated.

## 3. Channel Estimation with Interference Suppression

### 3.1. Interference Suppression in CIR

We perform LS estimation to Equation (3), and the CIR of frequency domain is denoted as ${\hat{H}}_{LS}^{\left(1\right)}\left(k,l\right)$

(4) $${\hat{H}}_{LS}^{\left(1\right)}\left(k,l\right)=\frac{Y(k,l)}{X(k,l)}=H\left(k,l\right)+\frac{J(k_{I},l)}{X(k,l)}+\frac{W(k,l)}{X(k,l)}.$$

It can be seen from Equation (4) that there are three categories of signals in the *l*th symbol in *k*th carrier: CIR, interference and noise. The existence of interference is uncertain, and the signal feature is unknown. System performance will be deteriorated with the interference incorrectly cancelled as AWGN [23]. It is necessary to detect and suppress the interference before noise cancellation.

Usually, NBI is added to partial subcarriers by high-powered signals. Thus, the receive signal power of the subcarriers that are interfered with will be higher than the others. As in CIR, the modulus of the interfered subcarriers will be higher than the others. On the basis of the feature, we introduced machine learning techniques into interference cluster. Because the interference does not necessarily exist in CIR, we proposed using the clustering of interfered subcarriers based on the global *K*-means algorithm [24]. The main thought of the *K*-means algorithm is to start from the data to be measured, first randomly generate *K* clusters, and then calculate the similarity between the data to be measured and the cluster center, and continuously update both the cluster center and the cluster in the iterative calculation until the convergence of the cluster center point is completed. For interference clustering, the set of subcarriers with no interference is denoted as cluster $C_{1}$, and the set of interfered subcarriers is denoted as cluster $C_{2}$. Therefore, interference detection is equal to the cluster of samples in CIR.

The steps of *K*-means interference clustering are as follows:

**Step 1.** The data to be measured is denoted as $m_{i}$ and $m_{i}\in \left\{\right|{\hat{H}}_{LS}^{\left(1\right)}\left(k,l\right)\left|\right\},i=1,2,\ldots ,N$. The cluster center of $C_{1}$ is defined as the mean of $m_{i}$, which is denoted as $m_{t}$:

(5) $$m_{t}=\frac{1}{N}\sum_{i=1}^{N}m_{i}.$$

**Step 2.** Euclidean distance is used to measure the similarity between $m_{t}$ and $m_{i}$. The calculation formula is given as Equation (6):

(6) $$d_{i,t}=m_{i}-m_{t}.$$

**Step 3.** The cluster center of $C_{2}$ is denoted as $m_{s}$, which represents the data of the longest distance from $m_{t}$ in Equation (6). The distance between $m_{s}$ and $m_{i}$ is calculated as Equation (7):

(7) $$d_{i,s}=m_{i}-m_{s}.$$

**Step 4.**$m_{i}$ will be classified by the distance calculated in Equations (6) and (7):

(8) $$\left\{\begin{array}{cc} m_{i}\in C_{1} & \mathrm{if}\;d_{i,t}<d_{i,s},\\ m_{i}\in C_{2} & \mathrm{if}\;d_{i,t}\geq d_{i,s}. \end{array}\right.$$

**Step 5.** The cluster center $m_{s}$ will be updated as the cluster $C_{2}$ was updated. Step 3 and Step 4 will be repeated until all data to be measured are classified. The calculation formula of $m_{s}$ updating is given as Equation (9):

(9) $$m_{s}=\frac{1}{Q}\sum_{i\in C_{2}}m_{i},$$

where *Q* is denoted as the number of samples in $C_{2}$.

According to the interference cluster scheme, the modulus of samples in $C_{2}$ are bigger than those in $C_{1}$. The power of a few subcarriers is singularly high for Q≪N. It is fair to hypothesize that the corresponding subcarriers have been interfered with. When *Q* is not much less than *N*, the corresponding subcarriers are not interfered with or the interference is not significant to the subcarriers. Nevertheless, the subcarriers may be subject to non-narrowband interference and it is not the scope of this study.

It is necessary to suppress interference for Q≪N. The simplest way to suppress interference on a subcarrier is zero force, which means to set the samples on the interfered subcarriers in CIR to zero. However, the zero members will also participate in data demodulation and output error soft bits, which will have the adverse effect to channel decoding and degrade the overall system performance. On the other hand, the channel estimation will be deteriorated by setting the samples on the interfered subcarriers to zero in CIR.

The CIR in interfered subcarriers can not reflect the fading characteristics of the subcarriers. It is assumed that the channel is stationary for a continuous spectrum transmission. Thereby, the difference between CIR in the interfered subcarriers and the adjacent subcarriers will be small. Hence, we propose to suppress interference by linear interpolation of the CIR of frequency domain. The interpolation algorithm is presented as Equation (10):

(10) $$H\left(k_{I},l\right)=\left(1-\frac{b}{a+b}\right){\hat{H}}_{LS}^{\left(1\right)}\left(k_{I}-a,l\right)+\frac{b}{a+b}{\hat{H}}_{LS}^{\left(1\right)}\left(k_{I}+b,l\right),$$

where *a* is the normal carrier at the left of the interfered subcarriers, and *b* is the normal carrier at the right of the interfered subcarriers. Both *a*, *b* will be at the left/right of the interfered subcarriers, which are at the far right/far left.

Through linear interpolation on the interfered subcarriers, $J(k_{I},l)$ in Equation (4) will be cancelled, and it will be rewritten as Equation (11):

(11) $${\hat{H}}_{LS}^{\left(2\right)}\left(k,l\right)=H^{\prime }\left(k,l\right)+\frac{W(k,l)}{X(k,l)}.$$

It must be noted that linear interpolation on the interfered subcarriers is to improve the accuracy of channel estimation, which can not restore original data on interfered subcarriers.

The *K*-means algorithm can achieve the local optimization in unsupervised learning [25]. On the premise of NBI, the *K*-means algorithm will be an effective scheme for interference detection. However, even if the signal component is misjudged as interference, the line interpolation over adjacent subcarriers will not have a serious negative impact on CIR.

### 3.2. Noise Cancellation in CIR

The noise term in Equation (11) can be cancelled in the CIR of time domain. To enhance resolution of the CIR of time domain, M=2N points Inverse Fast Fourier Transform (IFFT) is applied to ${\hat{H}}_{LS}^{\left(2\right)}\left(k,l\right)$, so we have Equation (12):

(12) $${\hat{h}}_{LS}^{\left(2\right)}\left(m,l\right)=h^{\prime }\left(m,l\right)+\frac{1}{M}\sum_{k=1}^{M-1}\frac{W(k,l)}{X(k,l)}e^{j2\pi \frac{km}{M}}.$$

Because the IFFT of W(k,l) is also approximately Gaussian, the noise of the time domain can be denoted as w(m,l). Equation (12) can be rewritten as:

(13) $${\hat{h}}_{LS}^{\left(2\right)}\left(m,l\right)=h^{\prime }\left(k,l\right)+w\left(m,l\right).$$

Because the envelop feature of ${\hat{h}}_{LS}^{\left(2\right)}\left(m,l\right)$ is known to the receiver, $h^{\prime }\left(k,l\right)$ and w(m,l) can be classified by the machine learning mechanism. We proposed the classification of CIR and noise based on the *K*-nearest neighbor (KNN) algorithm [26]. The mean thought of KNN is to set up a training sample for each category, and then find out *K*-nearest neighbors which have the greatest similarity with the data to be measured from all the training sample sets. The data to be measured will be classified into the category that is the majority of its neighbors.

According to the envelop feature of ${\hat{h}}_{LS}^{\left(2\right)}\left(m,l\right)$, which can be decomposed into two sets theoretically, one of the sets is denoted as $\tilde{S}$, which represents the set of modulus of signal components. Because of the leakage of CIR, $\tilde{S}$ exists at the beginning and end of ${\hat{h}}_{LS}^{\left(2\right)}\left(m,l\right)$:

(14) $$\tilde{S}=\left\{\right|{\hat{h}}_{LS}^{\left(2\right)}\left(m,l\right)||0\leq m\leq 2M_{CP}\}\cup \{|{\hat{h}}_{LS}^{\left(2\right)}\left(m,l\right)||M-M_{CP}\leq m\leq M\},$$

where the maximum multipath delay is denoted as $M_{CP}$.

The other set is denoted as $\tilde{W}$, which represents the set of modulus of noise components:

(15) $$\tilde{W}=\left\{\right|{\hat{h}}_{LS}^{\left(2\right)}\left(m,l\right)||2M_{CP}<m<M-M_{CP}\}.$$

For the noise components in $\tilde{S}$ realistically, we set up the training samples of signal component by the largest *P* samples in $\tilde{S}$. The initial set of training samples of signal component is denoted as $G_{1}$:

(16) $$\begin{array}{c} G_{1}=\left\{u_{i}|\left(u_{1}\geq u_{2}\geq \ldots \geq u_{P-1}\geq u_{P}\right)\wedge u_{i}\in \tilde{S}\right\},\\ {\hat{\mu }}_{1}=\frac{1}{P}\sum_{m=1}^{P}u_{m},\\ {\hat{\sigma }}_{1}=\frac{1}{P-1}\sum_{m=1}^{P}{\left|u_{m}-{\hat{\mu }}_{1}\right|}^{2}, \end{array}$$

where ${\hat{\mu }}_{1}$ and ${\hat{\sigma }}_{1}$ are the mean and variance of the elements in $G_{1}$.

According to the principle of 3σ in Gaussian distribution, the modulus of noise samples less than σ was a big probability. For there are signal components in $\tilde{W}$ realistically, we set up the noise training samples with modulus less than σ in $\tilde{W}$. The initial set of training samples of noise component is denoted as $G_{2}$:

(17) $$\begin{array}{c} G_{2}=\left\{v_{i}|\left(-\sigma <v_{i}<\sigma \right)\wedge v_{i}\in \tilde{W}\right\},\\ {\hat{\mu }}_{2}=\frac{1}{Q}\sum_{m=1}^{Q}v_{m},\\ {\hat{\sigma }}_{2}=\frac{1}{q-1}\sum_{m=1}^{Q}{\left|v_{m}-{\hat{\mu }}_{2}\right|}^{2}, \end{array}$$

where σ is the variance of the elements in $\tilde{W}$, ${\hat{\mu }}_{2}$ and ${\hat{\sigma }}_{2}$ are the mean and variance of the elements in $G_{2}$.

It can be seen from Equations (16) and (17) that the statistical properties of $G_{1}$ and $G_{2}$ are different. We should calculate the similarity by the standardized Euclidean distance, in order to eliminate the error by the distribution difference of samples.

The data to be measured is denoted as $q_{m}\in \left\{\right|{\hat{h}}_{LS}^{\left(2\right)}\left(m,l\right)\left|\right\},m=1,2,\ldots ,M.$ The steps of KNN classification are as follows.

**Step 1.** Calculate the standardized Euclidean distance between $q_{m}$ and all the samples in $G_{1}$ and $G_{2}$. $D_{m}$ is the set of all the calculation results:

(18) $$\begin{array}{c} d^{2}\left(q_{m},v_{i}\right)=\frac{{\left(q_{m}-v_{i}\right)}^{2}}{{\hat{\sigma }}_{2}^{2}},\\ d^{2}\left(q_{m},u_{j}\right)=\frac{{\left(q_{m}-u_{j}\right)}^{2}}{{\hat{\sigma }}_{1}^{2}},\\ D_{m}=\left\{d^{2}\left(q_{m},v_{i}\right)\right\}\cup \left\{d^{2}\left(q_{m},u_{j}\right)\right\}. \end{array}$$

**Step 2.** Sort $D_{m}$ according to ascending order and find out the first *k* elements. $T_{m}^{NN}$ is a set of first *k* elements, which mean that the *k* nearest neighbor of $q_{m}$, and $q_{m}$ will be classified by majority vote in $T_{m}^{NN}$:

(19) $$G\left(q_{m}\right)=\underset{G_{r}}{\arg\max}\sum_{t_{k}\in T_{m}^{NN}}\delta \left[G_{r}=G\left(t_{k}\right)\right],$$

where:

$G(q_{m})$ represents the classification of the training samples $t_{k}$,

$\delta [G_{r}=G\left(t_{k}\right)]$ is Kronecker Delta function,

r∈{1,2} represents the two classifications as $G_{1}$ and $G_{2}$.

**Step 3.** Update $G_{1}$ or $G_{2}$ according to $G(q_{m})$, and update the corresponding mean and variance.

Reiterate the three steps until all the samples in ${\hat{h}}_{LS}^{\left(2\right)}\left(m,l\right)$ have been classified. Then, we set all the samples to zero in $G_{2}$ for noise cancellation. Finally, we obtain the channel estimation as:

(20) $${\hat{h}}_{LS}^{\left(3\right)}\left(m,l\right)=h^{\prime }\left(k,l\right).$$

Signal components and noise components are independent of CIR in the time domain. The classifying error of KNN in two categories is presented in [27]:

(21) $$R_{NN}^{*}\leq \rho_{k}\left(R_{NN}^{*}\right)\leq \rho_{k-1}\left(R_{NN}^{*}\right)\leq \ldots \leq \rho_{2}\left(R_{NN}^{*}\right)\leq \rho_{1}\left(R_{NN}^{*}\right)=2R_{NN}^{*}\left(1-R_{NN}^{*}\right),$$

where $R_{NN}^{*}$ is Bayes probability of error, and *k* is the number of nearest neighbors.

It can be seen from Equation (21) that noise cancellation with the KNN algorithm will be close to the Bayes probability of error when the nearest neighbors *k* is big enough.

We have achieved interference suppression in the CIR of frequency domain by the *K*-means algorithm and noise cancellation in the CIR of time domain by the KNN algorithm. Although the original data on the interfered subcarriers can not be restored by the accurate channel estimation, the original data on the interfered subcarriers could be partially restored by channel decoding. Soft bits demodulated from interfered subcarriers should be identified in channel decoder in order to reduce the weight of these soft bits.

**Figure 2:**
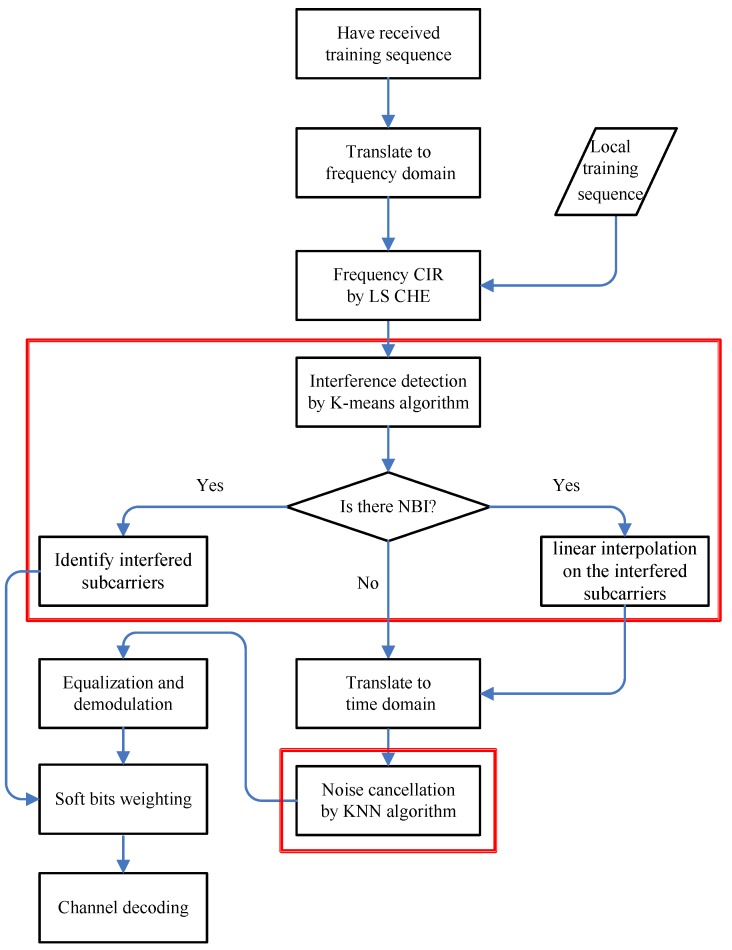
The integrated process of channel estimation with interference suppression.

The complete process of channel estimation with interference suppression is described in Figure 2. Steps in the red box are key contents of the process.

## 4. Complexity Analysis and Performance Simulation

In this section, we will evaluate the computational complexity and performance of the proposed channel estimation with traditional channel estimation. For easy description, the proposed channel estimation scheme is abbreviated as Discrete Fourier transform-KNN (DFT-KNN). One of the comparative schemes is the frequency domain windowed scheme, which is a typical scheme of traditional transform domain channel estimation, and abbreviated as DFT-WF [10]. The other comparative scheme is a discriminant analysis scheme, which is one of the earliest channel estimation schemes based on machine learning techniques, and abbreviated as DFT-DA [11].

### 4.1. Complexity Analysis

We evaluate the computational complexity of DFT-KNN with others by multiply-accumulate (MAC) and multiply-add (MA) times. The computation of the algorithms modules are shown in Table 1, where core operator represents data width in parallel computing.

According to the process shown in Figure 2, the amount of computation of DFT-KNN is 2641.92 MMAC (million MAC). There are two times of FFT transformation, one time LS estimation, one time noise cancellation by widowing in DFT-WF, and the amount of computation of DFT-WF is 2396.16 MMAC. There are two times of FFT transformation, one time LS estimation, one time noise cancellation by Mahalanobis distance in DFT-DA, and the amount of computation of DFT-WF is 2519.04 MMAC.

The sum of computation of interference detection and interference suppression in DFT-KNN is less than 125 MMAC. DFT-KNN achieves interference suppression with amazing low computation load. The computational complexity of DFT-KNN than that of DFT-WF increased by 12.7%, and the computational complexity of DFT-KNN than that of DFT-DA increased by 3.7%.

The current processors can provide 100 GMAC (giga MAC) computing power, such as ZYNQ, C66x, in which DFT-KNN can be easily implemented [28]. Therefore, the algorithm is useful for node equipment in CPSS.

### 4.2. Simulation Results

We evaluate the performance of DFT-KNN with others by simulation comparison. The communication system parameters of simulation are shown in Table 2, and the channel parameters are shown in Table 3. To compare the performance of channel estimation objectively, the receiver is assumed to be synchronized accurately.

#### 4.2.1. Performance without Interference

**Figure 3:**
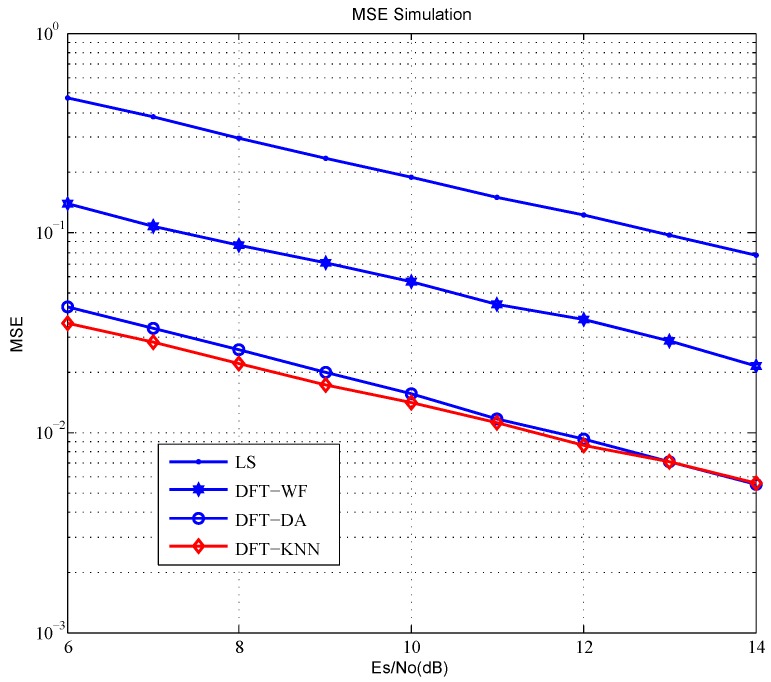
MSE of the channel estimation schemes.

Mean square errors (MSE) of the channel estimation schemes are shown in Figure 3. The performance increases obviously by noise cancellation in LS channel estimation. The MSE of DFT-WF outperforms LS over 4 dB. The MSE of DFT-DA and DFT-KNN outperform LS by 9 dB more or less.

In fact, all of these channel estimation schemes find the noise components by the modulus of the CIR of time domain. According to the envelop feature of CIR being known to the receiver, the signal and noise components are completely independent, and DFT-KNN that is based on a supervised learning algorithm can achieve Bayes minimum error. The frequency domain window has improved resolution of CIR of the time domain, but a lot of extra noise will be introduced to CIR by a filtering window function. It makes the performance gap of MSE between DFT-WF and DFT-KNN 5 dB more or less. DFT-DA is based on the Gaussian distribution of signal and noise components in CIR, but the signal and noise components obey the Rician distribution in the simulation, which makes the performance gap of MSE between DFT-DA and DFT-KNN by 0.5 dB more or less.

**Figure 4:**
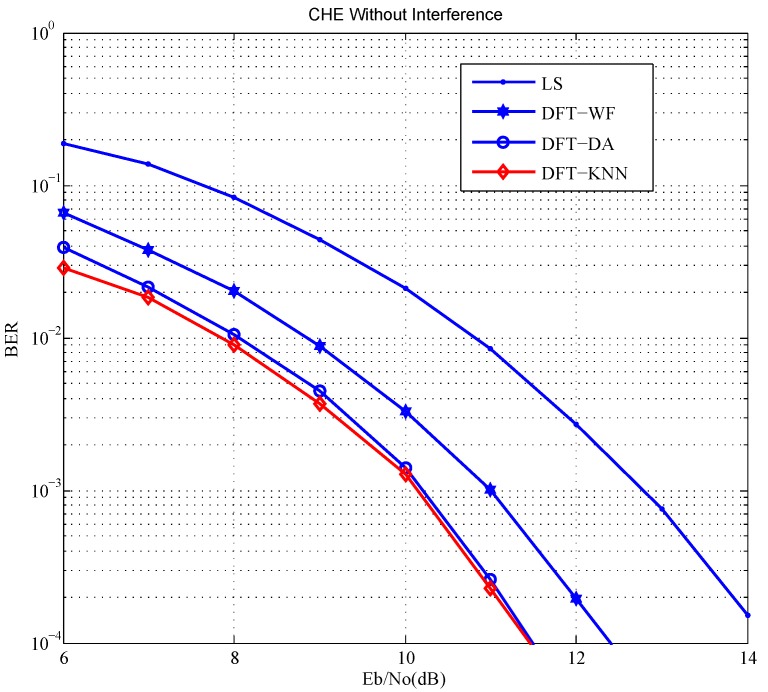
BER based on different channel estimation schemes.

The system bit error rate (BER) based on different channel estimation schemes are shown in Figure 4. Because of the performance difference in channel estimation schemes, the BER of DFT-WF outperforms LS close to 2 dB, the BER of DFT-DA outperforms LS by 2.5 dB more or less, the BER of DFT-KNN outperforms LS by 3 dB more or less. DFT-KNN has the best performance in the simulation.

#### 4.2.2. Performance under Interference

Table 4 describes the simulation scenes under different combinations of interference styles, jamming signal ratio (JSR) and interference suppression method.

**Figure 5:**
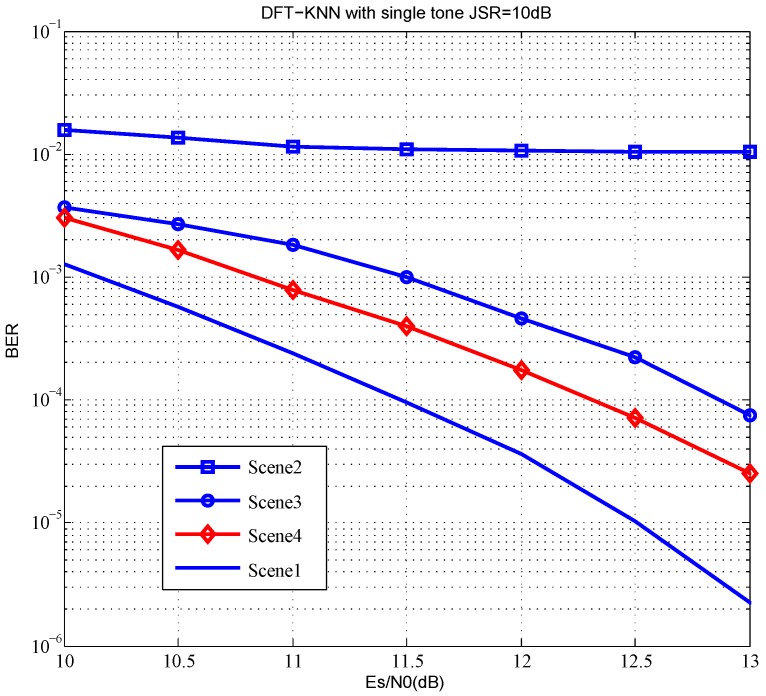
BER in different interference suppression schemes.

The system BER in different interference suppression schemes are shown in Figure 5. BER has a floor effect with nothing to do about the interference in the system. All of the transform domain channel estimation algorithms may have such a problem, including DFT-DA and DFT-WF. Linear interpolation on the interfered subcarriers outperforms zero force by 0.5 dB. Meanwhile, a BER performance loss of scene 4 which has JSR = 10 dB is within 1 dB compared to scene 1 that has no interference. It can be proved that DFT-KNN tends to be more effective in complex electromagnetic environments.

**Figure 6:**
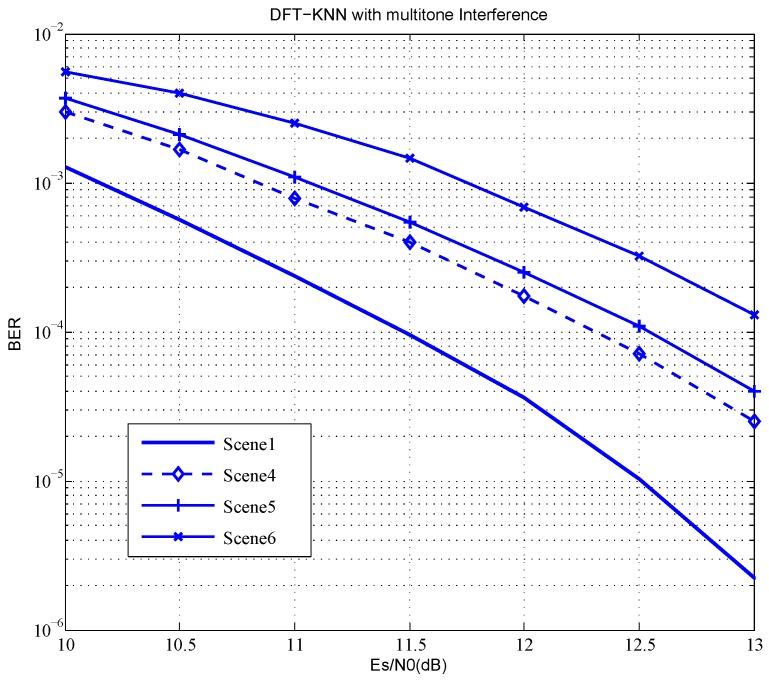
BER in different interference styles.

The system BER in different interference styles is shown in Figure 6. Although the interference power of a single subcarrier in multitone jamming is lower than that of single tone jamming, the interfered subcarriers will lead to a series of inaccurate soft bits, which will degrade the performance of the channel decoder. Hence, BER in multitone jamming is lower than that of single tone jamming. Even with the JSR up to 15 dB, BER performance loss of scene 6 is within 2 dB compared to scene 1 that has no interference. DFT-KNN is proven robust in complex electromagnetic environments.

## 5. Method Application

As an important component in CPSS, data serve as a carrier system connecting cyberspace, physical world and artificial world. Depending on the communication network, data in both the physical and artificial world are transmitted to cyberspace, which are analyzed and calculated in the cyberspace. The analytic results and decision making then give feedback to the physical and artificial world through the communication network. Thus, the stability and reliability of the communication network is the prerequisite for efficient CPSS operation.

When applied in telemedicine, based on various wearable device sensors, CPSS can continuously monitor physiological vital signs and transmit them for remote diagnosis. Interference in communication may cause distortion of data [29]. When proper data cannot be received by the control center in time, the quality of health monitoring is affected which may further lead to misdiagnosis and failure to provide timely treatment to the patients [30]. In CPSS-based intelligent transportation systems, the position of mobile phones and vehicles can be used to analyze the dynamic changes of traffic conditions, so as to carry out traffic flow control and traffic guidance. Similar applications require human beings, vehicles and roadsides to be connected via universal and ubiquitous networks enabled by wireless communications, such that they can act cooperatively. The coexistence and cooperation of the wireless communications give rise to interference coordination and resource allocation problems [31].

Due to the diversity of communication systems in CPSS, frequency spectrum coordination is becoming increasingly difficult. That interference suppression provides an alternative option for anti-interference.

**Figure 7:**
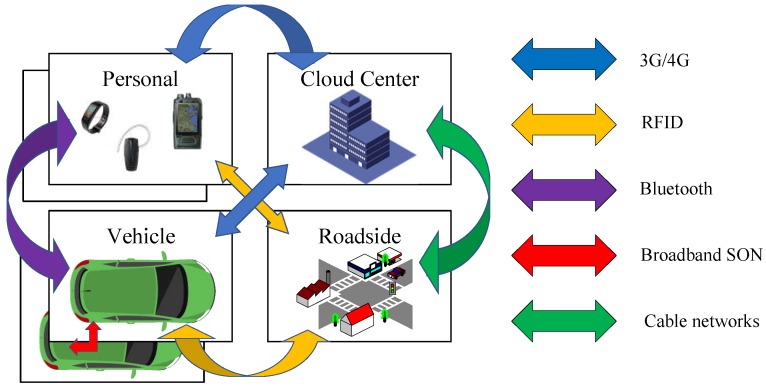
Communication systems in ICV.

We have applied the proposed scheme to an intelligent connected vehicle (ICV). As shown in Figure 7, the ICV established the connection with the cloud center through the public mobile communication network, such as 3 G, 4 G, 5 G, and established the connection with roadside and human through short-range communications such as RFID, bluetooth, and the connection with other vehicles via broadband self-organization networks (SON). Based on these communication systems, the resources on the clouds, roadsides and vehicles have been integrated seamlessly. By jointly considering the information from physical, social, and cyberspace, safety and optimal driving experience could be effectively realized.

**Figure 8:**
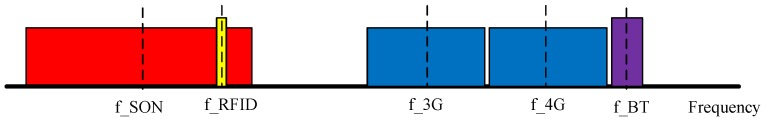
Spectrum division of the communication systems in ICV.

There are multiple wireless communication systems in such a small vehicle, and the wireless channel will be a complex electromagnetic environment of mixed radio signals. In our experimental ICV, RFID and SON happened to be in the same frequency spectrum (Figure 8). The mutual interference would appear when the two communication systems work simultaneously. In actual assessment, the loss rate of transmission in vehicle-to-vehicle reached 15% without interference suppression. After applying the proposed scheme in SON, which combined interference suppression in channel estimation, the loss rate of transmission in vehicle-to-vehicle showed no significant changes regardless of whether RFID is working concurrently or not.

## 6. Conclusions and Future Work

This study proposed a novel channel estimation with interference suppression, which used machine learning to meet the requirements of CPSS in complex electromagnetic environments.

The *K*-means algorithm was used for interference detection in CIR of frequency domain, and suppressed the interference with linear interpolation of interfered subcarriers. The KNN algorithm was used for noise cancellation in the CIR of time domain, which is close to Bayes probability of error. Both *K*-means in CIR of frequency domain and KNN in CIR of time domain have relatively lower complexity in channel estimation, which are compliant for nodes in CPSS. Using extensive simulations, the performance of the proposed scheme is proven superior to the traditional scheme. The proposed scheme is proven to work effectively in an interference scene and the negative effects of transmission in interference are under control.

Our future work will focus on the implementation and optimization of the scheme, hopefully helping to improve the transmission stability and reliability in CPSS. 

## Figures and Tables

**Table 1:** Computation of algorithm modules.

Algorithm Modules	Basis of Algorithm	Core Operator	Computation
FFT transformation	Radix-4	16b × 16b MAC	1013.76 M
LS estimation	Least square	16b × 16b MA	122.88 M
Interference detection	*K*-means	16b × 16b MAC	122.88 M
Interference suppression	Linear interpolation	16b × 16b MA	*negligible*
noise cancellation 1	KNN	16b × 16b MAC	368.64 M
noise cancellation 2	Windowing	16b × 16b MAC	245.76 M
noise cancellation 3	Mahalanobis distance	16b × 16b MA	368.64 M

**Table 2:** Transmission parameters.

Parameters	Specifications
Carrier frequency	400 MHz–700 MHz
Modulation type	16 QAM ${}^{a}$
Transmission rate	23.56 Mbps
Subcarrier number	1024
Subcarrier spacing	15 kHz
Channel codes	Turbo

${}^{a}$ QAM, Quadrature Amplitude Modulation.

**Table 3:** Channel parameters.

Tap Number	Average Power (dB)	Relative Delay (ns)
0	−3	0
1	0	200
2	−2	600
3	−6	1600
4	−8	2400
5	−10	5000

**Table 4:** Simulation scenes with different interference styles.

Simulation Scene	Interference Style	JSR	Interference Suppression Method
Scene 1	No interference	–	–
Scene 2	Single tone	JSR = 10 dB	With nothing done
Scene 3	Single tone	JSR = 10 dB	Zero force
Scene 4	Single tone	JSR = 10 dB	Linear interpolation
Scene 5	multitone with 10 subcarriers	JSR = 10 dB	Linear interpolation
Scene 6	multitone with 10 subcarriers	JSR = 15 dB	Linear interpolation
